# Modulated contact frequencies at gene-rich loci support a statistical helix model for mammalian chromatin organization

**DOI:** 10.1186/gb-2011-12-5-r42

**Published:** 2011-05-10

**Authors:** Franck Court, Julie Miro, Caroline Braem, Marie-Noëlle Lelay-Taha, Audrey Brisebarre, Florian Atger, Thierry Gostan, Michaël Weber, Guy Cathala, Thierry Forné

**Affiliations:** 1 Institut de Génétique Moléculaire de Montpellier (IGMM), UMR5535 CNRS, Universités Montpellier 1 et Montpellier 2. 1919, Route de Mende, 34293 Montpellier Cedex 5, France; 2 Current address: INSERM U827, Laboratoire de Génétique des Maladies Rares, IURC, 64, avenue du Doyen G Giraud, 34093 Montpellier Cedex 5, France

## Abstract

**Background:**

Despite its critical role for mammalian gene regulation, the basic structural landscape of chromatin in living cells remains largely unknown within chromosomal territories below the megabase scale.

**Results:**

Here, using the 3C-qPCR method, we investigate contact frequencies at high resolution within interphase chromatin at several mouse loci. We find that, at several gene-rich loci, contact frequencies undergo a periodical modulation (every 90 to 100 kb) that affects chromatin dynamics over large genomic distances (a few hundred kilobases). Interestingly, this modulation appears to be conserved in human cells, and bioinformatic analyses of locus-specific, long-range *cis-*interactions suggest that it may underlie the dynamics of a significant number of gene-rich domains in mammals, thus contributing to genome evolution. Finally, using an original model derived from polymer physics, we show that this modulation can be understood as a fundamental helix shape that chromatin tends to adopt in gene-rich domains when no significant locus-specific interaction takes place.

**Conclusions:**

Altogether, our work unveils a fundamental aspect of chromatin dynamics in mammals and contributes to a better understanding of genome organization within chromosomal territories.

## Background

Within the interphasic cell nucleus, the mammalian genome, packed into the chromatin, is spatially restrained into specific chromosomal territories [1,2] and is distributed in at least two spatial compartments: one enriched in active genes and open chromatin [3-7] and the other containing inactive and closed chromatin [4,7,8]. It was recently proposed that, at the megabase (Mb) scale, chromosome territories consist of a series of fractal globules [4]. However, below that scale, and beyond the simple nucleosomal array, the basic structural landscape of the chromatin in living cells remains enigmatic.

At the supranucleosomal level (approximately 10 to 500 kb), it is largely accepted that one essential determinant in relation to gene expression and other chromosomal activities is chromatin looping [9]. However, because of technological limitations, access to this level of chromatin organization remains problematic [10]. From this perspective, the advent of the Chromosome Conformation Capture (3C) assay [11,12] represents a decisive technological and scientific breakthrough since it permits the identification of long-range *cis *and *trans *chromatin interactions in their native genomic context. Subsequently, several 3C-based methods have been developed that allow the unbiased large-scale identification of such interactions [4,7,13-16]. Noticeably, the use of a population-based approach like the 3C-real-time quantitative PCR (qPCR) protocol [17,18], combined with appropriate algorithms for accurate data normalization [19], provides a powerful quantitative method that allows high-resolution analysis (on the kilobase scale) of the average contact frequencies between distant genomic regions within a locus. This information is particularly interesting as contact frequencies essentially depend on constraints that the chromatin may undergo at that scale. Constraints resulting from locus-specific interactions are easily identified in 3C-qPCR experiments since they appear as local peaks where the interaction frequency is at least four to five times higher than the surrounding collision levels [17,19]. Furthermore, they are detected only in some experiments targeting specific regulatory sequences within a given locus. On the contrary, intrinsic constraints, resulting from fundamental characteristics of the chromatin (compaction, flexibility, basic non-linear shape), are expected to have a similar impact on contact frequencies at many sites and numerous loci.

Here, using a 3C-qPCR approach [17], we determined random collision frequencies within interphase chromatin at several mouse loci. We demonstrate that, in the absence of significant locus-specific interactions, several gene-rich domains of the chromatin display modulated contact frequencies in both mouse and human, thus revealing the existence of an unexpected intrinsic constraint. We propose that this constraint results from a preferential non-linear shape that the chromatin tends to adopt and show that the observed modulations can be described by polymer models as if, at these loci, the chromatin was statistically shaped into a helix.

## Results

### Several mouse gene-rich loci display modulation of contact frequencies

**Figure 1:**
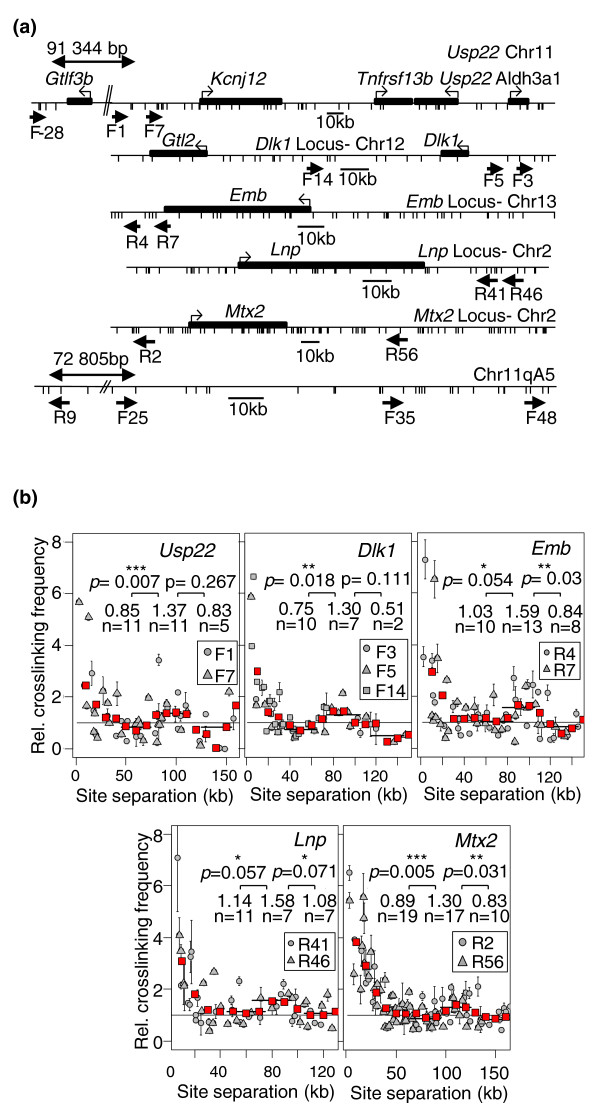
**Random collision frequencies at five mouse gene-rich loci**. **(a) **Maps of mouse loci investigated. Genes are indicated by full boxes and promoters by thick black arrows. The scale bar indicates the size of 10 kb of sequence. The names of the loci and chromosomal location are indicated above each map. The HindIII (*Usp22*, *Emb*, *Lnp*, *Mtx2 *and 11qA5 gene-desert loci) or EcoRI (*Dlk1 *locus) sites investigated are indicated on the maps. Arrows indicate the positions of the primers used as anchors in 3C-qPCR experiments. **(b) **Random collision frequencies at five mouse gene-rich loci. Locus names are indicated above each graph. Random collision frequencies were determined by 3C-qPCR in the 30-day-old mouse liver at the indicated anchor sites (for further details see Materials and methods). They were determined in three independent 3C assays each quantified at least in triplicate and the data were normalized as previously described [19]. Error bars are standard error of the mean of three independent 3C assays. Grey circles, triangles or squares are data points obtained from distinct genomic sites as indicated on the graphs. In each graph, red squares represent the floating mean (20-kb windows, shift of 10 kb). *P*-values (Mann-Whitney *U*-test) account for the significance of the differences observed between the higher and the lower points of the floating mean. They were calculated from the values of the average random collision frequencies in a window of 30 kb around these points (values indicated in the figure) (One asterisk indicates a *P*-value < 0.1 and > 0.05; double asterisks a *P*-value < 0.05 and > 0.01 and triple asterisks a *P*-value < 0.01).

To focus on the interphase chromatin, we worked on preparations of cell nuclei from postnatal mouse livers [20,21], and to minimize potential interference of locus-specific long-range interactions, we restricted our analysis to mouse loci where no significant local peaks could be detected in 3C-qPCR experiments. As previously suggested for its human ortholog [22], the mouse *Usp22 *(*Ubiquitin carboxyl-terminal hydrolase 22*) locus, on chromosome 11, displays such characteristics. Two intergenic HindIII sites (F1 and F7 in Figure 1a) were separately used as anchors to determine interaction frequencies with other HindIII sites found throughout this locus. As expected, for site separations lower than 35 kb, random collision frequencies decrease with increasing site separations (Figure 1b, upper-left panel). However, a floating mean analysis of these data (red squares in Figure 1b) indicated a stabilization of random collision frequencies around 60 kb and a surprising increase for higher site separations, reaching a maximum for distances around 100 kb. Indeed, between these two positions, the mean interaction frequency (0.85 versus 1.37, respectively) increases very significantly (*P *= 0.007, Mann-Whitney *U*-test). We then investigated four additional gene-rich loci that displayed no evidence for long-range specific interactions in the postnatal mouse liver: the *Dlk1 *(*Delta-like 1 homologue*) locus on chromosome 12 [19,23], the *Lnp *(*Limb and neural patterns/Lunapark*) and *Mtx2 *(*Metaxine 2*) loci on chromosome 2, and the *Emb *(*Embigin*) locus on chromosome 13 (Figure 1a). Interestingly, similar modulation in random collision frequencies was shown at all four loci (Figure 1b). In conclusion, for eleven intergenic sites (anchors) distributed in five loci and four distinct mouse chromosomes, one can always observe that random collision frequencies increase for site separations around 80 to 110 kb. Therefore, this modulation reflects some intrinsic constraints resulting from fundamental properties of the chromatin (compaction, flexibility, basic non-linear shape) rather than a locus-specific interaction.

**Figure 2:**
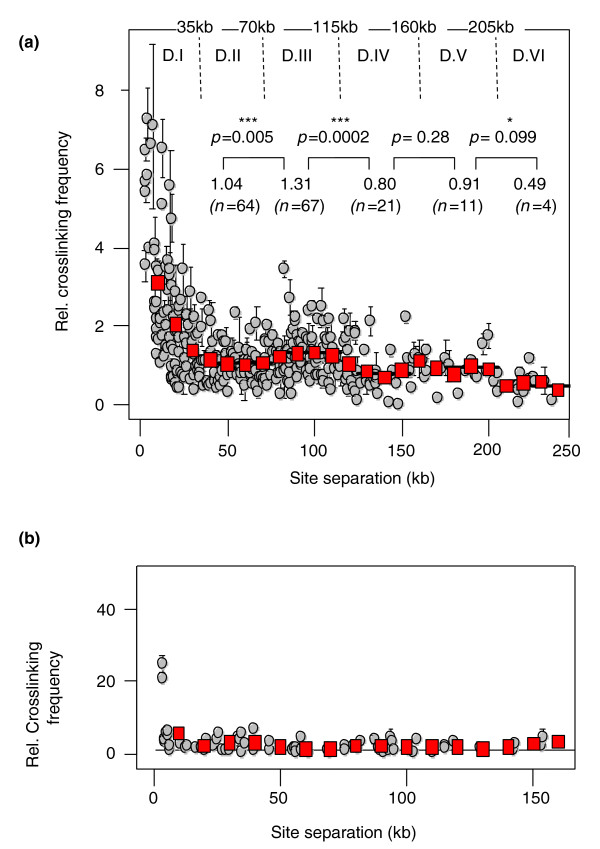
**Random collision frequencies in gene-rich and gene-desert regions**. **(a) **Experimental data obtained for mouse gene-rich regions (shown in separate graphs in Figure 1b) have been plotted into a single graph. A few data points at separation distances above 150 kb, which were omitted in Figure 1b, are included. Statistical analyses were performed on the floating mean (red squares) as explained in Figure 1b. The dashed lines delimit supranucleosomal domains (D.I to D.VI) that encompass separation distances where random collision frequencies are alternatively lower and higher: 0 to 35 kb (domain I), 35 to 70 kb (domain II), 70 to 115 kb (domain III), 115 to 160 kb (domain IV), 160 to 205 kb (domain V) and 205 to 250 kb (domain VI). **(b) **Random collision frequencies were determined by 3C-qPCR at four sites (R9, F25, F35 and F48; Figure 1) located in an AT-rich/gene-desert region located on mouse chromosome 11. Red squares represent the floating mean (20-kb windows, shift of 10 kb). Error bars are standard error of the mean (the triple asterisks indicate a *P*-value < 0.01).

Since this modulation was similar at all loci investigated, we plotted all the data into a single graph (Figure 2a). Statistical analyses indicated a significant increase of random collision frequencies for site separations around 100 kb compared to those around 60 kb (*P *= 0.005, Mann-Whitney *U*-test), followed by a very significant decrease between 100 and 140 kb (*P *= 0.0002, Mann-Whitney *U*-test). Very interestingly, random collision frequencies stabilized between 140 and 180 kb before finally dropping for distances above 180 kb (*P *= 0.099, Mann-Whitney *U*-test; Figure 2a). This observation suggests that a second significant modulation for separation distances may occur around 180 kb and raise the possibility that these modulations occur with a periodicity of approximately 90 kb.

To assess this periodicity, we needed to examine random collision frequencies for larger site separations. This was made possible by adding a primer extension step to the 3C protocol (see Materials and methods). We then repeated experiments at the anchor site F1 of the *Usp22 *locus and investigated a novel genomic site (F-28) located one potential modulation away (91.3 kb upstream from site F1 and 109.9 kb from site F7) (Figure 1a). These experiments validated our observations in two separate biological samples (embryonic day 16.5 and adult mouse liver) for site separation distances as far as 340 kb, revealing three consecutive modulations with a periodicity of about 90 to 100 kb (Additional file 1). Noticeably, as expected, site F-28, located 90 to 100 kb (one modulation) upstream of sites F1 and F7, displays a similar modulation in contact frequencies, confirming, once again, that this phenomenon is unlikely to result from site-specific interactions.

We conclude that several gene-rich mouse loci display an unexpected 90-kb modulation that affects contact frequencies over large genomic distances. To simplify further statistical analyses, we decided to describe this 90-kb modulation as consecutive supranucleosomal domains encompassing separation distances where random collision frequencies alternate between high and low values (Figure 2a).

### Contact frequencies at a mouse gene-desert locus

Previous 3C studies in yeast [11] and human [14] indicated strong differences for chromatin dynamics between GC-rich and AT-rich/gene-poor loci [24]. To assess whether such differences also exist in the mouse, we investigated four genomic sites (anchors) located within a gene-desert/AT-rich region of the 11qA5 chromosomal band (Figure 2b). Consistent with previous work in human [14], we found that random collision frequencies decrease dramatically for short site separations, reaching very low basal random collision levels for sites separated by only 5 to 6 kb. Opposite to gene-rich regions, however, no significant increase was observed for large site separations. We conclude that chromatin dynamics in gene-desert domains is radically different from that observed in intergenic portions of gene-rich domains, with random collisions frequencies noticeably decreasing much more rapidly for shorter genomic distances.

### Modulated contact frequencies at gene-rich loci are conserved in human chromatin

To assess whether modulated contact frequencies of gene-rich domains could be detected in human chromatin, we used published 'Chromosome Conformation Capture Carbon Copy' (5C) data obtained at the human *β*-globin locus [13] from experiments where only residual (very weak) locus-specific interactions were detected. Statistical analysis revealed a significant increase of random collision frequencies for site separations around 100 kb (*P *= 0.022, Mann-Whitney *U*-test) followed by a very significant decrease for larger site separations (*P *= 0.0003, Mann-Whitney *U*-test) (Additional file 2). Therefore, the 90-kb modulation observed for random collision frequencies at several mouse gene-rich loci appears to be conserved at the human *β*-globin locus.

### Genomic consequences of modulated contact frequencies

**Figure 3:**
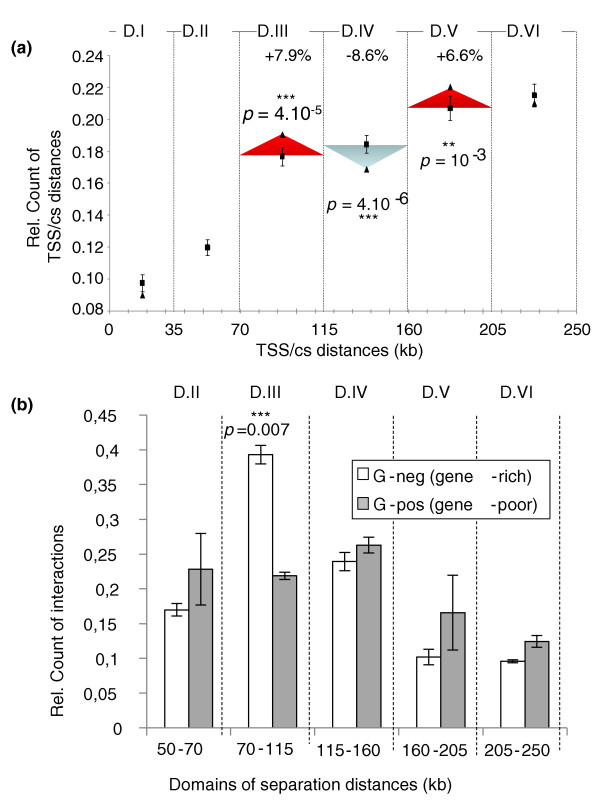
**Influence of modulated random collision frequencies on long-range interactions and mammalian genome evolution**. **(a) **Separation distances between conserved sequences (cs) and transcription start sites (TSS) of co-expressed mouse genes were determined as explained in the Materials and methods section. Black triangles depict the relative count of separation distances obtained for each supranucleosomal domain. Black squares indicate the mean of relative counts obtained from 30 random samples of genes. Error bars represent the 95% confidence intervals for randomization. Separation distances are significantly over-represented in domains III and V (+7.9% and +6.6%, respectively) while they are significantly under-represented in domain IV (-8.6%) (*P*-values of *t*-tests are indicated on the graph). **(b) **Histogram depicting the relative counts of *cis-*interactions in human GM06990 or K562 cells (Hi-C experiments from [4]) occurring in Giemsa-negative (gene-rich regions, white bars) or Giemsa-positive (gene-poor regions, gray bars) bands. For each set, the number of interactions was counted in each supranucleosomal domain (as defined in Figure 2a). Counts in each domain were normalized against the total number of sequence-tags counted over all domains (D.I to D.VI). Error bars represent standard error of the mean of two Hi-C experiments. The *P-*value indicated on the figure was obtained from a *t-*test (double asterisks indicate a *P*-value < 0.05 and >0.01, and triple asterisks a *P*-value < 0.01).

Modulations in contact frequencies, as observed here for gene-rich regions, should have fundamental implications for gene regulation and mammalian genome evolution. Indeed, if, as demonstrated in this work, the frequency of random collisions does not regularly decrease according to genomic distances but displays a periodical modulation, then *cis*-regulatory sequences that (for mechanistic reasons) should interact together over long distances will tend to accumulate at preferred relative separation distances where the collision dynamics is fundamentally the most prone to such contacts. According to this proposal, *cis-*interacting sequences should position into supranucleosomal domain I (less than 35 kb) or domain III (around 90 kb), and eventually in domain V (around 180 kb), since the higher basal collision levels are found in these domains. Using the READ Riken Expression Array Database [25], we identified 130 mouse genes that display strong co-expression patterns with at least one other gene located less than 400 kb away in *cis *(see Materials and methods) and showed that, around such co-expressed genes, conserved sequences are significantly over-represented in both domain III (+7.9%) and domain V (+6.6%) (*P *= 4 × 10^-5 ^and 1 × 10^-3^, respectively, *t*-tests from randomizations) (Figure 3a). The number of conserved sequences is close to a random distribution in domains I and II but shows a significant under-representation (-8.6%; *P *= 4 × 10^-6^, *t*-test) in domain IV (between the first and second modulations) where the lower random collisions frequencies were observed. We conclude that, as a predicted consequence of our findings, conserved intergenic sequences of clustered co-expressed genes are significantly over-represented within supranucleosomal domains III and V corresponding to the first and second modulations of random collision frequencies.

Interestingly, recent genome-wide mapping of chromosomal interactions in human by Hi-C experiments also provides direct experimental validation of our proposal. Indeed, these data confirm that long-range interactions in Giemsa-negative bands, containing gene-rich regions, are favored for site separations around 90 kb (domain III) relative to Giemsa-positive bands, which are gene-poor regions (Figure 3b). Therefore, both bioinformatic analyses and genome-wide Hi-C experiments support the predicted consequences of a 90-kb modulation and suggest that this phenomenon underlies the chromatin dynamics of a significant number of gene-rich loci in mammals.

### The statistical helix model

We reasoned that the modulations of contacts frequencies observed at several gene-rich loci may reflect a preferential statistical shape that the chromatin tends to adopt when no strong locus-specific interactions take place. Since this constraint appears to be independent of the genomic position at all five gene-rich loci investigated, this preferential non-linear shape should possess a long-range translational symmetry. This led us to postulate that this statistical shape may correspond to a simple helix organization.

The dynamics of chromatin has been successfully modeled in yeast [11,24] using a Freely Jointed Chain/Kratky-Porod worm-like chain model [26]. This model is given in Equation 1 [24], which expresses the relationship between crosslinking frequency *X*(*s*) (in mol × liter^-1 ^× nm^3^) and site separation *s *(in kb):(1)

The *β *term represents the number of Kuhn's statistical segments and depends on polymer shape. Equations 2a and 2b (see Materials and methods) provide the *β *terms used for linear and circular polymers, respectively. For a polymer folded into a circular helix, we developed the following *β *term (see Materials and methods):(5)

where *D *is the diameter of the helix (in nm) and *P *its step (in nm). In the above equations, *S *is the length of the Kuhn's statistical segment in kb, which is a measure of the flexibility of the chromatin, and *k *is the crosslinking efficiency, which reflects experimental variations. The linear mass density *L *is the length of the chromatin in nm that contains 1 kb of genomic DNA.

**Figure 4:**
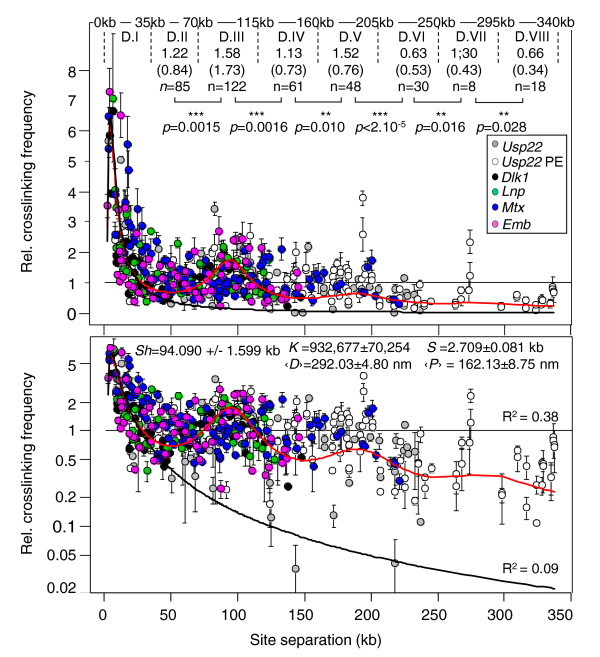
**Fitting the statistical helix polymer model to random collision frequencies quantified at mouse gene-rich loci**. 3C-qPCR data shown in Figure 2a and Additional file 1 (*Usp22*PE) were compiled into a single graph (upper panel). Error bars are standard error of the mean. The dashed lines delimit supranucleosomal domains as defined in Figure 2a. The graph shows the best fit analyses obtained with the linear polymer model (Equations 1 and 2a; black curve) or the statistical helix model (Equations 1 and 5; red curve). Correlation coefficients (*R*^2^) are indicated in the lower panel, which shows the same graph where collision frequencies are represented in a logarithmic scale. Best fit parameters for the statistical helix model are indicated within the graph (lower panel) and have been used to calculate the expected theoretical means of random collision frequencies for each supranucleosomal domain (numbers in brackets in upper panel), which are in good agreement with the means obtained from the experimental data (values indicated above the expected means). *P*-values (Mann-Whitney *U*-test) account for the significance of the differences observed between the experimental means of two adjacent domains. One can note, amongst the experimental points, a few outliers. To minimize the weight of these data points, we chose a non-parametric statistical test (double asterisks indicate a *P*-value < 0.05 and > 0.01 and triple asterisks a *P*-value < 0.01).

Using Equation 1 and the appropriate *β *terms, we fitted our experimental data to three polymer models. The linear model fits appropriately only for site separations lower than 35 kb (domain I; black line in Figure 4, lower panel). By setting an apparent circular constraint (*c *= 110.515 ± 2.028 kb), the circular polymer model [11] better fits the experimental data but only for site separations lower than this apparent circular constraint *c *(that is, below 110kb) (Additional file 3). Finally, the statistical helix model provides a valid description over the entire range of genomic distances investigated (0 to 340 kb; *R*^2 ^= 0.38; red line in Figure 4). Importantly, this finding shows that modulated contact frequencies observed at mammalian gene-rich loci can be described as if the chromatin was statistically shaped into a helix for which we estimated the structural parameters: diameter *D *= 292.03 ± 4.80 nm and step *P *= 162.13 ± 8.75 nm (Figure 4). Noteworthy, the estimated length of the statistical segment *S *= 2.709 ± 0.081 kb, indicates that the mammalian chromatin is more flexible than its yeast counterpart, for which a value of *S *= 4.7 ± 0.45 kb was obtained for GC-rich regions [24]. These parameters allow calculation of the length of DNA folded into one turn of this statistical helix: *Sh *= 94.090 ± 1.599 kb (see Materials and methods).

**Figure 5:**
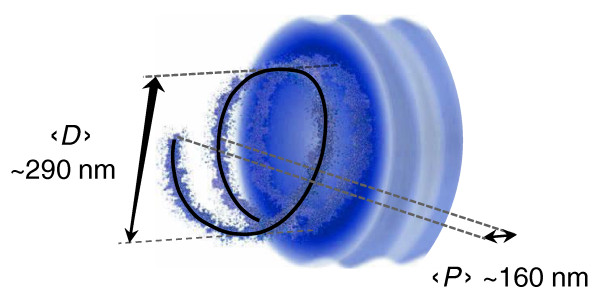
**The statistical helix model**. The statistical helix model that we propose in this study (Equations 1 and 5) suggests that, in the absence of strong locus-specific interactions, some gene-rich domains of the mammalian chromatin tend to adopt a helix shape. This helix is averaged over the whole population of cells analyzed (5 million nuclei in each 3C sample) and thus more likely represents a statistical shape arising from the global dynamics of the chromatin than a fixed organization. It is characterized by a mean diameter 〈*D*〉 and mean step 〈*P*〉, and it thus likely corresponds with the place where the probability of finding the chromatin at a given *t *time is the highest (black helical curve).

It is important to stress that the shape of the chromatin described by these parameters is averaged over the whole population of cells analyzed (5 million nuclei in each 3C sample) and thus is more likely to represent a statistical shape arising from the global dynamics of the chromatin than a fixed organization (Figure 5).

## Discussion

This work reveals that some gene-rich regions of the mouse and human genomes display modulation of their contact frequencies. Several lines of evidence indicate that this modulation arises from an intrinsic constraint rather than from locus-specific constraints. Firstly, for a given locus, a similar 90-kb modulation is observed at several genomic sites assayed. For example, at the *Dlk1 *locus it occurs at site F3 and sites F5 (9 kb away from F3) and F14 (62.7 kb away); at the *Usp22 *locus, it takes place at site F-28 as well as sites F1 (91.4 kb away) and F7 (109.9 kb away). Secondly, this 90-kb modulation was found at five distinct gene-rich loci located on four different mouse chromosomes. Finally, using published 5C data [13], we found a very similar modulation at the human *β*-*globin *locus in cells where very weak interactions were found. Interestingly, this modulation was not revealed in previous 3C experiments that we, and many others, performed in mouse or human. There are at least two reasons why this phenomenon went unnoticed. Firstly, the amplitude of the modulation is very weak and could only be significantly revealed when a relatively large number of experimental points were obtained from a highly quantitative method and combined together into a single graph after accurate normalization of the data [19]. Secondly, at many gene-rich loci (see, for example, [14]), strong locus-specific interactions (above four times the local random collision level) take place, which very likely perturb this modulation. However, as observed in this work (outliers in Figure 4) or in GM06990 cells for the human *β*-globin locus [13] (Additional file 2), modulation can be perceived despite some residual and weak locus-specific *cis*- or *trans*-interactions (below three to four times the local random collision level). Interestingly, this modulation is not a simple consequence of gene expression *per se *since RT-qPCR analysis indicated that, in the samples investigated (30-day-old mouse liver), some loci are completely repressed (*Dlk1 *locus), or display very low expression levels (*Emb *and *Lnp *loci), while others contain expressed genes (*UspP22 *and *Mtx2 *loci) (Additional file 4). However, according to our modeling, the statistical helix would be in a slightly more 'open' configuration at the expressed loci (with a diameter *D *of about 303.92 ± 6.55 nm and a step *P *of 177.38 ± 12.05 nm), compared to silent loci (*D *= 278.83 ± 7.65 nm and *P *= 149.20 ± 13.67 nm) (Additional file 5). Nevertheless, these differences are minor and the statistical helix model is valid in both situations.

To what extent does this phenomenon apply to substantial parts of mammalian genomes? Our work suggests that gene-rich regions of the mammalian chromatin display modulated contact frequencies while no modulation could be evidenced in gene-poor regions (Figure 2b). As previously discussed, direct experimental detection of such modulations requires finding cellular systems where no strong locus-specific interactions occur. This is an important caveat that is particularly difficult to circumvent at many gene-rich loci that we may wish to investigate. In this work, the modulation could be observed at only five mouse and one human loci. Therefore, it remains difficult to speculate on whether such a phenomenon may apply to a substantial part of gene-rich domains, or whether it is rather limited to few loci. Clearly, however, both bioinformatic analyses and genome-wide mapping of chromatin interactions [4] indicate that this phenomenon may underlie the dynamics of a significant number of locus-specific interactions in gene-rich domains of mammalian chromatin (Figure 3).

As previously mentioned, one consequence of modulated contact frequency is that long-range interacting *cis*-regulatory sequences will undergo constraints that will tend to accumulate them within specific supranucleosomal domains where the collision dynamics is fundamentally the most appropriate for contacts. This property may explain the peculiar arrangements of genes and *cis-*regulatory elements observed at several important mammalian loci, such as the 'global control region' (GCR) at the mouse *Hoxd *(*Homeobox d*) locus, which is located at one or two modulations away from the genes that it regulates. It was suggested that 'the GCR would have concentrated, in the course of evolution, several important enhancers, due to an intrinsic property to work at a distance' [27]. The modulation of contact frequencies revealed in this work represents one such intrinsic property that may contribute to enhancer clustering in mammals.

Our work suggests that modulated contact frequencies arise from an intrinsic constraint that applies to the chromatin. This led us to wonder about the nature of this constraint and to propose that it may result from a preferential statistical shape that the chromatin tends to adopt in gene-rich regions when no strong locus-specific interactions take place. This hypothesis is supported by the finding that modulated contact frequencies can be described by polymer models as if, in these regions, the chromatin was statistically shaped into a helix (Figure 4). Interestingly, by using 3C data obtained in the yeast *Saccharomyces cerevisiae *[24], we showed that the statistical helix model may also be valid for GC-rich (but not AT-rich) domains of the yeast genome (Additional files 6 and 7).

One consequence of folding the chromatin into a helix-shaped structure is that the volume it occupies increases dramatically. This increase can be estimated by calculating the volumetric mass density (*Vs*) of the statistical helix. In mammals, *Vs *= 1.02 × 10^5 ^± 0.05 × 10^5 ^nm^3^/kb (or 0.0098 ± 0.0005 bp/nm^3^; estimated from Equation 6 given in the Materials and methods section and best fit parameter shown in Figure 4). This can be compared to the estimated volumetric mass density *V *of the postulated 30-nm chromatin fiber: *V *= 6.8 × 10^3 ^nm^3^/kb (calculated from Equation 6 with *D *= 30 nm; 〈*R*〉 = 9.6 nm and *s *= 1 kb). Therefore, the folding of a putative 30-nm chromatin fiber into a statistical helix would result in a 15.00 ± 0.73-fold increase (*Vs/V*) of the volume that it occupies. Finally, if the entire diploid genome had a helical chromatin organization as shown in Figure 5, it would occupy a volume of about 610 μm^3 ^(the volume occupied by such a helix encompassing two times 3 × 10^9 ^bp), which is higher than the volume of a regular mammalian nucleus (approximately 520 μm^3 ^for a nuclear diameter of 10 μm). Therefore, in addition to the helix-shaped organization described above, other types of dynamic folding should exist that achieve higher levels of chromatin compaction. This hypothesis is supported by our finding showing that the dynamics of random collisions in gene-desert regions is completely different to that observed in gene-rich domains.

The pioneering work of Ringrose *et al*. [28] demonstrated that chromatin behaves like a linear polymer at short distances. This work was based on quantitative comparison of *in vivo *recombination events and was limited to short site separation distances (less than 15 kb). Our work suggests that the upper limit for such linear polymer models may occur, in gene-rich regions, for separation distances around approximately 35 kb (supranucleosomal domain I; Figure 4). For higher genomic distances, spanning at least 340 kb (Figure 4), the statistical helix polymer model describes accurately the dynamics of the chromatin. What is the upper limit of validity for this model? We know that, at a larger scale, the chromatin is confined within the limited space of the chromosome territory [2,29]. This 'chromosomal territory constraint' will necessarily impact on the accuracy of the statistical helix polymer model to describe chromatin dynamics. Cell imaging techniques have suggested that polymer models are incompatible with spatial distance measurements obtained for genomic separations over 4 Mbp [30,31]. Therefore, the upper limit should lie somewhere between 340 kb and 4 Mbp. Based on the biophysical parameters provided in Figure 4, we calculated how, in interphasic cells, the spatial distances should vary as a function of genomic site separations and compared the resulting values to those measured in fluorescence *in situ *hybridization (FISH) experiments. For separation distances below 1 Mb, spatial distances predicted from the statistical helix model (red curve in Additional file 8) are fully compatible with the distances measured in FISH experiments (data points in Additional file 8) [32]. However, above 1 Mb, the statistical helix model does not fit with the experimental data and, therefore, the upper limit of validity of this model appears to reside at separation distances around 1 Mb. This suggestion is in agreement with the recent comprehensive mapping of chromosomal interactions in the human genome (Hi-C experiments) showing that, above the megabase scale, the chromatin adopts a 'fractal globule' conformation [4]. In line with modeling approaches pioneered by Dekker and colleagues [11,24], our work suggests that, below the megabase scale, chromatin dynamics within such globules can be accurately described by appropriate polymer models. We can reasonably expect that the increasing sensitivity of both cell imaging and 3C-derived techniques will soon help us to assess the validity of this approach, thus enlightening one of the last remaining 'mysteries' of mammalian genome organization.

## Conclusions

In this work, we have discovered an unexpected 90- to 100-kb modulation of contact frequencies at gene-rich loci of mammalian chromatin. We show that this modulation has important implications for genome evolution and we provide an original model that suggests that the modulation may result from a fundamental statistical helix shape that the chromatin tends to adopt when no significant locus-specific interactions are taking place. Altogether, our work contributes to a better understanding of the fundamental dynamics of mammalian chromatin within chromosomal territories.

## Materials and methods

### Mouse breeding

All experimental designs and procedures were in agreement with the guidelines of the animal ethics committee of the French 'Ministère de l'Agriculture'.

### 3C-qPCR/SybGreen assays

The 3C-qPCR assays were performed as previously described [17] with a few important modifications that increased the efficiency of the 3C assays four-fold, thus allowing real-time PCR quantifications of 3C products using the SybGreen technology instead of TaqMan probes used in previous work [17,19]. The 3C-qPCR method [17] was modified as follows. Step 2: 5 × 10^6 ^nuclei were crosslinked in 1% formaldehyde. Step 8: added 5 μl of 20% (w/v) SDS (final 0.2%). Step 10: added 50 μl of 12% (v/v) Triton X-100 diluted in 1 × ligase buffer from Fermentas (40 mM Tris-HCl pH7.8, 10 mM MgCl_2_, 10 mM DTT, 5 mM ATP). Step 13: added 450 U of restriction enzyme (EcoRI for the *Dlk1 *locus or HindIII for the other loci). Step 16: incubated 30 minutes at 37°C; shake at 900 rpm. Step 34: additional digestions were performed using BamHI for the *Dlk1 *locus and StyI for the other loci. Step 39: adjusted 3C assays with H_2_O to 25 ng.μl^-1^. 3C products were quantified (during the linear amplification phase) on a LighCycler 480 II apparatus (Roche, Basel, Switzerland); 10 minutes at 95°C followed by 45 cycles 10 s at 95°C/8 s at 69°C/14 s at 72°C) using the Hot-Start Taq Platinum Polymerase from Invitrogen (Carlsbad, California, USA) (10966-34) and a standard 10 × qPCR mix [33] where the usual 300 μM dNTP were replaced with 1,500 μM of CleanAmp dNTP (TEBU 040N-9501-10). Standards curves for qPCR were generated from BACs (Invitrogen) as previously described [17]: RP23 55I2 for the *Usp22 *locus; RP23 117C15 for the *Dlk1 *locus; and a subclone derived from RP23 3D5 for the gene-desert region. For 3C-qPCR analyses of site F-28 at *Usp22 *locus, a PCR product encompassing 733 bp around site F-28 was generated from genomic DNA (FA4 gccatactcagccacagggac and RA2 cctgatctcacgaatcaccctc). This PCR product (0.1 μg) was mixed with 3.4 μg of the RP23 55I2 BAC before HindIII digestion and ligation to generate standard curves. Data obtained from these experiments are included in Additional file 9 (gene-rich loci) or Additional file 10 (gene-desert locus). 3C-qPCR primer sequences are given in Additional file 11. The number of sites analyzed in each experiment were as follows: *Usp22 *locus, for anchor sites F1 and F7, 34 and 40 sites, respectively; *Dlk1 *locus, for anchor sites F14/F5 and F3, 23/17 and 9 sites, respectively; *Emb *locus, for anchor sites R4 and R7, 31 and 30 sites, respectively; *Lnp *locus, for anchor sites R41 and R46, 27 and 25 sites, respectively; *Mtx2 *locus, for anchor sites R2 and R56, 52 sites for each anchor; and for the gene-desert locus, for anchor sites R9/F25/F35 and F48, 36/40/40 and 38 sites, respectively.

### Primer extension

For each biological sample and each extension primer (1F, cagtccagtgagacacatggttg; FA1, gttaaacccacagggcaagagc), six reactions were performed, pooled, purified with a QiaQuick PCR purification kit and diluted in H_2_O at 12.5 ng.μl^-1^. Each reaction was done as follows: 0.1 μM of extension primer was added to a 10-μl reaction containing 1 × qPCR mix [33] and 1 μl of highly concentrated 3C assay (containing about 200 to 300 ng of genomic DNA). Primers were extended by the Hot-Start Taq Platinum polymerase (Invitrogen) in a LightCycler apparatus (3 minutes at 95°C followed by 45 cycles 1 s at 95°C/5 s at 70°C/15 s at 72°C). Amplified 3C products were quantified by qPCR as explained above. Data obtained from these experiments are included in Additional file 9.

### RT-qPCR quantification

Total RNA extraction and RT-qPCR quantification were performed as previously described [20,21] using Superscript III reverse transcriptase (Invitrogen; 150 U for 45 minutes at 50°C).

### Supranucleosomal domains

Supranucleosomal domains (D.I to D.VI) were defined from statistical analyses (Mann-Whitney U tests) performed on data shown in Figure 2a. They encompass separation distances where random collision frequencies are alternatively lower and higher: 0 to 35 kb (domain I), 35 to 70 kb (domain II), 70 to 115 kb (domain III), 115 to 160 kb (domain IV), 160 to 205 kb (domain V) and 205 to 250 kb (domain VI).

### Mathematical methods

We used the Freely Jointed Chain/Kratky-Porod worm-like chain model [26]. This model is given in Equation 1 (Equation 3 of [24]]), which expresses the relationship between the crosslinking frequency *X*(*s*) (in mol × liter^-1 ^× nm^3^) and the site separation *s *(in kb):(1a)

with, for a linear polymer:(2a)

In Equation 1, *S *is the length of the Kuhn's statistical segment in kb, which is a measure of the flexibility of the chromatin, and *k *is the efficiency of crosslinking, which reflects experimental variations. The linear mass density *L *is the length of the chromatin in nm that contains 1 kb of genomic DNA. For the following analyses, we used a value *L *= 9.6 nm/kb [26] estimated from a packing ratio of 6 nucleosomes per 11 nm of chromatin in solution at physiological salt concentrations, corresponding to a nucleosome repeat length of about 190 bp, as found in mammalian cell lines. By introducing parameter *c *giving the 'apparent circle size' in kb into the *β *term of Equation 2a, Dekker *et al*. [11] derived a model (Equation 2b) that describes the dynamics of interactions within a circular polymer:(2b)

The *β *term in Equation 1 corresponds to the number *n *of Kuhn's statistical segments [26], which is directly related to the average spatial distance between the sites 〈*R*〉 in nm and the length of the statistical segment *S *as given in Equation 3:(3)

Interestingly, by setting appropriately the 〈*R*〉 parameter in Equation 3 and using the resulting *β *term in Equation 1, one can simulate spatial constraints that 'fold' the intrinsically linear polymer. Such modifications help us to model the dynamics of random collisions within a chromatin that possesses higher levels of organization. For a linear polymer, the average spatial distance 〈*R*〉 is directly linked to site separation *s *as given in Equation 4a:(4a)

and thus substitution of Equation 4a in Equation 3 yields the *β *term given in Equation 2a. For a circular polymer, the average spatial distance 〈*R*〉 can be linked to site separation *s *by introducing the previously described [11] apparent circular constraint *c *as given in Equation 4b:(4b)

and thus substitution of Equation 4b in Equation 3 yields the *β *term given in Equation 2b.

For a polymer folded into a circular helix the average spatial distance 〈*R*〉 (in nm) is related to site separation *s *(in kb), to the mean diameter *D *of the helix in nm and the mean step *P *in nm as given in Equation 4c:(4c)

Substitution of Equation 4c in Equation 3 yields the *β *term given in Equation 5:(5a)

Finally, the *β *term given in Equation 5 can be used in Equation 1 to provide a model that describes random collisions within a circular helix polymer. (Note that, for *P *= 0, Equation 5 describes a circularized polymer of size *D *and when both *P *= 0 and *D *tend to infinity the equation is able to describe a linear polymer). The length of one turn on the statistical helix *Sh *was calculated from the best-fit curve (Figure 4) by applying the second derivative method.

The volumetric mass density of the supranucleosomal chromatin *Vs *was calculated from Equation 6:(6)

where 〈*R*〉 corresponds to Equation 4c.

### Best-fit analyses

Best-fit analyses were implemented under the R software [34]. We used the '*nls *object' (package *stats *version 2.8.1), which determines the nonlinear (weighted) least-squares estimates of the parameters of nonlinear models.

### Bioinformatics and statistical analyses

Contact frequencies at the human *β*-globin locus in the EBV-transformed lymphoblastoid cell line GM06990 were downloaded from [13] (Supplemental Tables 6 and 7). These 5C data were normalized using our previously published algorithm [19] and compiled into a graph (Additional file 2).

Co-expressed genes were selected from the READ Riken Expression Array Database [25], which contains the relative expression levels of 16,259 transcripts in 20 mouse tissues. Housekeeping genes, which tend to accumulate in clusters [35] and are co-expressed but do not necessarily share *cis*-acting regulatory elements, have been excluded. According to the criteria defined by Ferrari and Aitken [36], housekeeping genes were considered as those having a *P*-value > 0.5. The resulting database contained 11,701 genes. We then retained only genes for which expression data were available for at least 15 tissues and selected gene pairs separated by less than 400 kb. This database, containing 6,619 genes, was used for identification of clustered co-expressed gene pairs and randomizations (see below).

For each possible gene pair, co-expression levels were determined by calculating the Pearson correlation coefficient (*r*) from their relative expression levels in at least 15 tissues. Co-expressed genes were defined as those having either similar (*r *≥ 0.8) or opposite (*r *≤ -0.8) tissue-specific expression patterns. This finally provided a set of 130 strongly co-expressed/co-regulated genes. We then determined the relative site separations between the transcriptional start sites of these co-regulated genes and conserved intergenic sequences. Conserved sequences were downloaded from the mouse genome (July 2007 assembly, filter 0.9, no overlap with UCSC Genes) on the UCSC server. We limited our analysis to a maximal separation distance of 250 kb covering the six previously defined supranucleosomal domains (Figure 2a). In order to obtain a database of conserved sequences that is significantly enriched in shared regulatory elements, we removed conserved sequences that are located in transcription units or promoter regions (less than 3 kb from a transcriptional start site). Finally, we counted site separation distances included in each domain and each count was normalized to the total site separation distances counted (over 250 kb). To evaluate the tendencies toward over- or under-representation of site separations in each domain, we randomly extracted 130 genes from the initial database and calculated site separation distances of conserved sequences, which were counted and normalized as mentioned above. This randomization was repeated 30 times. Normal distribution was checked for counts in each domain (Shapiro-Wilk tests). We then calculated the 95% confidence interval (*E*) from the following equation:

where *t *is the *t*-student variable as read in the Student's table for a degree of freedom of 29 and an alpha risk factor of 0.5 (*t *= 2.04), *μ *is the mean number of counts, *σ *is the standard deviation and *N *is the number of randomizations performed (here 30). Error bars represent the 95% confidence interval for counts in each domain.

Hi-C data used in Figure 3b are from published experiments [4]: Gene Expression Omnibus accession numbers [GSM455137] (sequencing of [GM06990] cells-lane1), [GSM455138] (sequencing of [GM06990] cells-lane2), [GSM455139] (sequencing of K562 cells-lane1) and [GSM455140] (sequencing of K562 cells-lane2). For each of the four datasets, we selected all the pairs of sequence tags located on the same chromosome and removed those located on distinct chromosomes (that is, we removed *trans*-interactions). Pairs of sequence-tags were classified by chromosome. We extracted the positions of all HindIII and NcoI sites from the human genome (hg18). Restriction fragments were numbered and, for each restriction fragment, we specified the positions of the 5' and 3' ends. The downloaded positions of the tags were replaced by the position of the corresponding restriction site. For this operation we used the restriction fragment numbers provided in the downloaded files. Direction '0' corresponds to a restriction site located at the 3' end of the restriction fragment (sense reading of the sequence-tag) while direction '1' corresponds to the 5' end (antisense reading of the sequence-tag). We then assembled datasets generated from lanes 1 and 2 of each experiment. We extracted from the UCSC server the positions of chromosomal bands (Giemsa-negative and Giemsa-positive; hg18). We selected all pairs of sequence-tags for which both partners are located within the same chromosomal band (to remove long-range/inter-band interactions). Data were pooled into two separate sets: a first set corresponding to all pairs of sequence-tags located in Giemsa-negative bands and a second one corresponding to pairs of sequence tags located in Giemsa-positive bands (threshold above 50, that is, g-pos-100, g-pos-75 and g-pos-50; see UCSC server). For each set, we selected interactions that are represented by at least four pairs of sequence tags (multiple pairs of sequence tags for identical interaction partners) and calculated for each interaction the separation distance between the restriction sites (using the positions previously calculated as described above). In each set (Giemsa-negative and Giemsa-positive), the number of interactions were counted in each supranucleosomal domain (as defined in Figure 2a) and this number was normalized to the total number of interactions counted in all domains (D.I to D.VI). Data are presented in a histogram (Figure 3b) that provides, for each domain, a comparison between the counts of interactions in Giemsa-negative and Giemsa-positive sets.

## Abbreviations

3C: Chromosome Conformation Capture; BAC: bacterial artificial chromosome; FISH: fluorescence *in situ *hybridization; qPCR: real-time quantitative polymerase chain reaction.

## Competing interests

The authors declare that they have no competing interests.

## Authors' contributions

FC improved the 3C protocol, performed 3C-qPCR experiments, developed an algorithm for 3C data processing, contributed to development of the mathematical models and performed bio-informatics analyses. JM and CB contributed to the design of the study and performed 3C-qPCR experiments. MNLT performed 3C-qPCR experiments. AB performed bio-informatics analyses. FA developed the primer extension step and performed 3C-qPCR experiments. TG contributed to bio-informatics analyses and performed statistical tests. MW developed best fit analyses and edited the manuscript. GC conceived of the study, performed 3C-qPCR experiments and edited the manuscript. TF conceived of and designed the study, contributed to the development of the mathematical models, performed best fit analyses and wrote the manuscript. All authors read and approved the final manuscript.

## Supplementary Material

Additional file 1 **Random collision frequencies in gene-rich regions for large separations distances**. Random collision frequencies were determined by 3C-qPCR after a primer extension step (see Materials and methods) at two *Usp22 *genomic sites (sites F1 and F-28) (Figure 1a) in liver samples from 16.5-days-post-coitus embryos (grey data points) or 30-day-old mice (white data points). Data analysis was as described in the legend of Figure 1b. Red squares represent the floating mean (45-kb windows, shift of 22.5 kb). We determined the higher and the lower points of the floating mean for site separations above 40 kb and calculated the average random collision frequencies (values are indicated in the figure) of sites located 40 kb around these points (horizontal black bars). *P*-values (Mann-Whitney *U*-test) account for the significance of the differences observed between these averages. Error bars are standard error of the mean. Click here for file

Additional file 2 **Collision frequencies at the human *β-globin *locus**. Collision frequencies at the human *β-globin *locus (a gene-rich region on chromosome 11p15.4) were obtained from several published 5C experiments performed in GM06990 cells, an EBV-transformed lymphoblastoid cell line where this locus is not expressed and where only a very weak/residual interaction was detected (Supplemental Tables 6 and 7 in [13]). Data from each experiment were normalized according to a previously published algorithm [19] and plotted into a single graph. Statistical analyses were performed as explained in the legend of Figure 1b. Click here for file

Additional file 3 **Fitting the circular polymer model to mouse gene-rich loci**. The circular polymer model (Equations 1 and 2b) was fitted to 3C-qPCR data obtained at gene-rich loci. The best fit curve is shown in red and best fit parameters are as follows: *R*2 = 0.50 with *K *= 725,785 ± 66,540; *S *= 2.515 ± 0.092 kb; *c *= 110.515 ± 2.028 kb. The black curve depicts the best fit obtained with the linear polymer model (Equations 1 and 2a; *R*2 = 0.18). Click here for file

Additional file 4 **Gene expression at loci investigated by 3C-qPCR**. Total RNA from 30-day-old mouse liver was prepared and mRNA levels were determined by RT-qPCR relative to *Gapdh *mRNA level. The *Usp22*, *LnP *and *Mtx2 *genes were found to be expressed. Very low levels of expression were found for the *Gtlf3b*, *Aldh3a2 *and *Emb *genes. The other genes (*Kcnj12*, *Tnfref13b*, *Gtl2*, *Dlk1 *and *HoxD13*) are fully repressed. Click here for file

Additional file 5 **Random collisions at silent versus expressed loci**. Data points represent collision frequencies determined at silent (*Dlk1*/*Emb*/*Lnp*; black circles) or expressed (*Usp22*/*Mtx2*; red circles) loci. Best fit of the statistical helix model (Equations 1 and 5) was performed for each dataset (black curve = silent loci; red curve = expressed loci). The values of best fit parameters for each data set are indicated in the graph. Both the diameter (*D*) and the step (*P*) of the helix are larger in the expressed loci compared to the silent ones. Click here for file

Additional file 6 **Fitting the statistical helix model to the yeast *Saccharomyces cerevisiae *genome**. In order to test whether a statistical helix organization may be valid for other organisms, we fitted the statistical helix polymer model to the 3C data obtained in the yeast *S. cerevisiae *[24]. For both AT-rich and GC-rich regions (Additional file 7a and 7b, respectively), correlation coefficients (*R*2 = 0.82 and 0.80, respectively) were similar to those obtained from published models (*R*2 = 0.81 and 0.79, respectively) [24]. For AT-rich regions, consistent with previous findings [24], the statistical helix model predicts a linear polymer organization (Additional file 7a). However, data obtained in GC-rich domains are fully compatible with a statistical helix organization. Compared to mammals, chromatin dynamics in yeast can be described as a statistical helix that would have a slightly smaller diameter (212.62 ± 31.73 nm) but a much wider step (310.94 ± 54.86) (Additional file 7b). Finally, using these best-fit parameters and Equation 4c, we calculated how, according to this statistical helix model, the spatial distances should vary as a function of genomic site separations. We found that spatial distances calculated from the statistical helix model are in good agreement with those measured in high-resolution FISH analyses performed in living yeast cells (Additional file 7c) [37]. Therefore, the statistical helix model may also be valid to describe chromatin dynamics in GC-rich domains of the *S. cerevisiae *genome. Click here for file

Additional file 7 **Fitting the statistical helix model to the yeast *Saccharomyces cerevisiae *genome**. Data published by Dekker for the yeast *S. cerevisiae *[24] were normalized using the previously published algorithm [19] and the statistical helix polymer model (Equations 1 and 5 was fitted to normalized data. **(a) **For AT-rich regions, consistent with previous findings [24], the statistical helix model (red curve) predicted a linear polymer organization (black curve). In this case, the best fit values obtained for the diameter *D *and the step *P *are not relevant, as indicated by large standard deviations. **(b) **In GC-rich regions, the statistical helix model (red curve), fits with a distended helical shape. Best-fit parameters are indicated above the graph. They were calculated using a linear mass density of 11.1 nm/kb [11]. The black curve depicts the best fit of the linear polymer model and the green curve the best fit of the circular polymer model. Note that the lengths of the statistical fragments obtained from the statistical helix model (*S *= 6.060 ± 0.519 kb and 4.558 ± 0.503 kb for AT-rich and GC-rich domains, respectively) are compatible with the parameters previously obtained with the linear or circular polymer models (*S *= 6.4 ± 0.34 kb and 4.7 ± 0.45 kb, respectively) [24]. **(b) **Using the best-fit parameters obtained for the yeast *S. cerevisiae *(b), we calculated the expected mean spatial distances (in nm) for increasing site separation distances (0 to 140 kb) for both the statistical helix (Equation 4c; red curve) and the linear polymer (Equation 4a; black curve) models. The experimental spatial distances (in nm) obtained by Bystricky *et al*. (Table 1 and Supplementary Table of [37]) from high-resolution FISH experiments were plotted into this graph (open squares, adjusted average distances; black diamonds, average peak distances). The statistical helix model is in good agreement with these experimental data. Click here for file

Additional file 8 **An upper limit of validity for the statistical helix model**. Expected spatial distances (in nm) were calculated as a function of increasing genomic distances (in kb) using either Equation 4a (linear polymer model, black curve, with *L *= 9.6 nm/kb) or Equation 4c and the biophysical parameter given in Figure 4 (statistical helix model, red curve). Dashed lines represent the expected deviations due to standard errors on the measured biophysical parameters (Figure 4). Details about mathematical equations are given in the Materials and methods section. Data points (blue diamonds) depict spatial distances measured by FISH experiments as reported by van den Engh *et al*. [32]. These data points were obtained from a gene-rich chromosomal region containing the Huntington disease locus. Click here for file

Additional file 9 **3C-qPCR dataset for gene-rich regions**. Click here for file

Additional file 10 **3C-qPCR dataset for the gene-desert region**. Click here for file

Additional file 11 **3C-qPCR primers**. Click here for file
