# Supplementary material for: Modulated contact frequencies at gene-rich loci support a statistical helix model for mammalian chromatin organization
Source: Genome Biol. 2011 May 10;12(5):R42. doi: 10.1186/gb-2011-12-5-r42 (PMC3219965; doi:10.1186/gb-2011-12-5-r42)
Supplement: Additional file 1 — Random collision frequencies in gene-rich regions for large separations distances. Random collision frequencies were determined by 3C-qPCR after a primer extension step (see Materials and methods) at two Usp22 genomic sites (sites F1 and F-28) (Figure 1a) in liver samples from 16.5-days-post-coitus embryos (grey data points) or 30-day-old mice (white data points). Data analysis was as described in the legend of Figure 1b. Red squares represent the floating mean (45-kb windows, shift of 22.5 kb). We determined the higher and the lower points of the floating mean for site separations above 40 kb and calculated the average random collision frequencies (values are indicated in the figure) of sites located 40 kb around these points (horizontal black bars). P-values (Mann-Whitney U-test) account for the significance of the differences observed between these averages. Error bars are standard error of the mean. [file gb-2011-12-5-r42-S1.PDF]

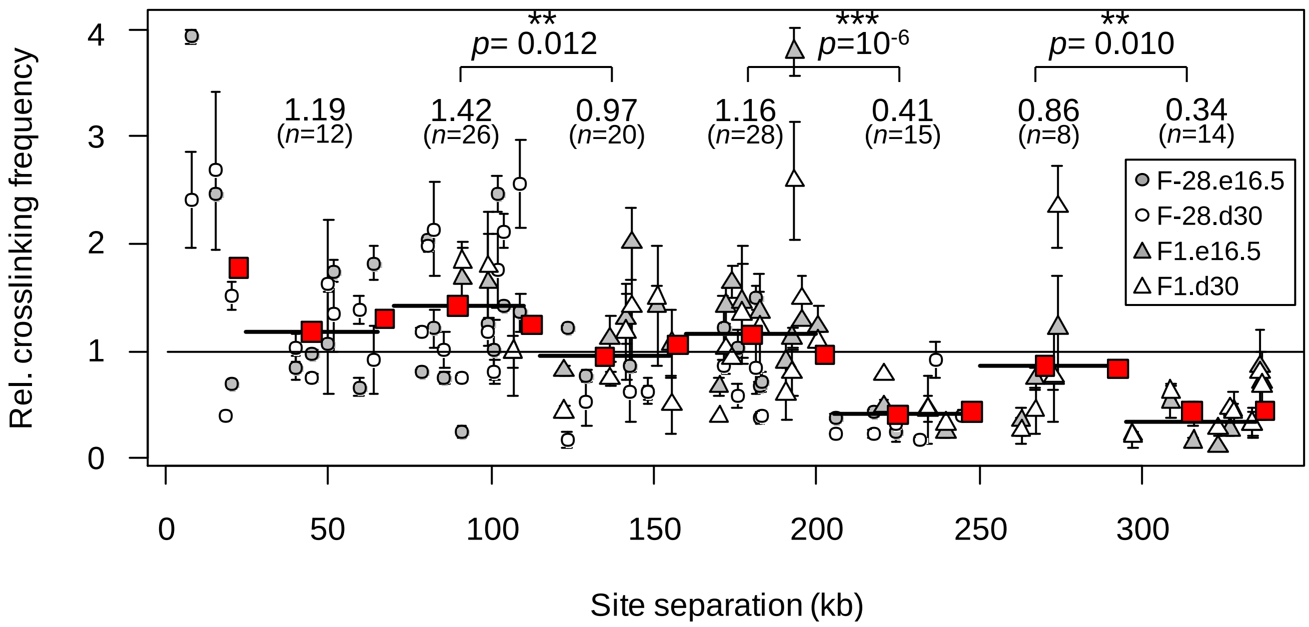

Additional data 1. Random collision frequencies in gene-rich regions for large separations distances.

Random collision frequencies were determined by 3C-qPCR after a primer extension step (see Methods) at two *Usp22* genomic sites (sites F1 & F-28) (Fig.1A) in liver samples issued from 16.5 dpc embryos (grey data points) or 30 days old mice (white data points). Data analysis was as described in the legend of Fig1B. Red squares represent the floating mean (45kb windows, shift of 22.5kb). We determined the higher and the lower points of the floating mean for site separations above 40kb and calculated the average random collision frequencies (values are indicated in the figure) of sites located 40kb around these points (horizontal black bars). *p*-values (Mann-Whitney *U*-test) account for the significance of the differences observed between these averages. Error bars are s.e.m.
