# Supplementary material for: Modulated contact frequencies at gene-rich loci support a statistical helix model for mammalian chromatin organization
Source: Genome Biol. 2011 May 10;12(5):R42. doi: 10.1186/gb-2011-12-5-r42 (PMC3219965; doi:10.1186/gb-2011-12-5-r42)
Supplement: Additional file 2 — Collision frequencies at the human β-globin locus. Collision frequencies at the human β-globin locus (a gene-rich region on chromosome 11p15.4) were obtained from several published 5C experiments performed in GM06990 cells, an EBV-transformed lymphoblastoid cell line where this locus is not expressed and where only a very weak/residual interaction was detected (Supplemental Tables 6 and 7 in [13]). Data from each experiment were normalized according to a previously published algorithm [19] and plotted into a single graph. Statistical analyses were performed as explained in the legend of Figure 1b. [file gb-2011-12-5-r42-S2.PDF]

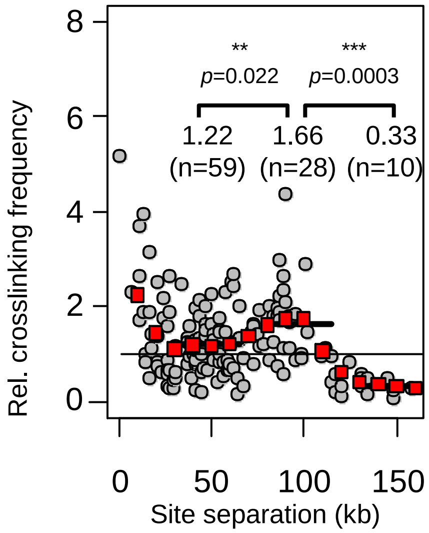

Additional data 2. Collision frequencies at the human *β-globin* locus.

Collision frequencies at the human *β-globin* locus (a gene-rich region on chromosome 11p15.4) were obtained from several published 5C experiments performed in GM06990 cells, an EBV-transformed lymphoblastoid cell line where this locus is not expressed and where only a very weak/residual interaction was detected (supplemental tables 6&7 of ref. [13]). Data from each experiment were normalized according to a previously published algorithm [19] and plotted into a single graph. Statistical analyses were performed as explained in the legend of Fig.1B.
