# Supplementary material for: Modulated contact frequencies at gene-rich loci support a statistical helix model for mammalian chromatin organization
Source: Genome Biol. 2011 May 10;12(5):R42. doi: 10.1186/gb-2011-12-5-r42 (PMC3219965; doi:10.1186/gb-2011-12-5-r42)
Supplement: Additional file 3 — Fitting the circular polymer model to mouse gene-rich loci. The circular polymer model (Equations 1 and 2b) was fitted to 3C-qPCR data obtained at gene-rich loci. The best fit curve is shown in red and best fit parameters are as follows: R2 = 0.50 with K = 725,785 ± 66,540; S = 2.515 ± 0.092 kb; c = 110.515 ± 2.028 kb. The black curve depicts the best fit obtained with the linear polymer model (Equations 1 and 2a; R2 = 0.18). [file gb-2011-12-5-r42-S3.PDF]

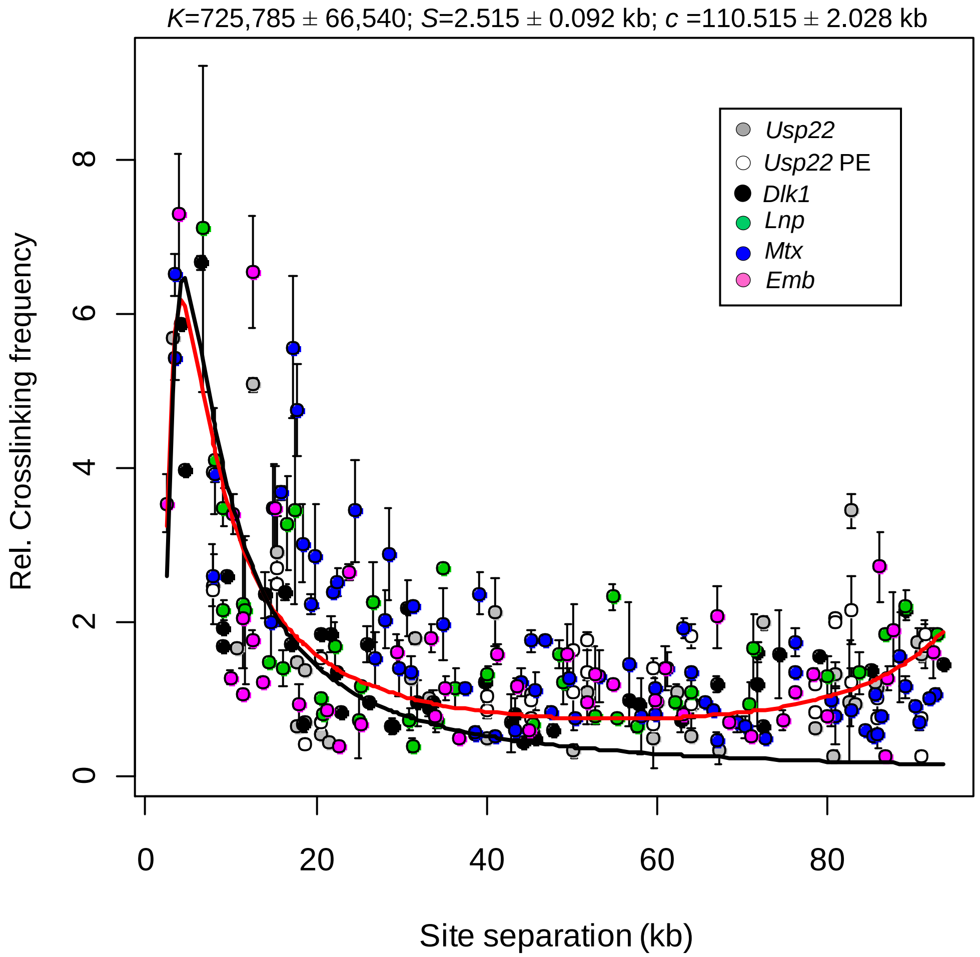

Additional data 3. Fitting the circular polymer model to mouse gene-rich loci. The circular polymer model (Eq.1 & 2b) was fitted to 3C-qPCR data obtained at gene-rich loci. The best fit curve is shown in red; best fit parameters are as follows: R^2=0.50 with *K*=725,785 ± 66,540; *S*=2.515 ± 0.092 kb; *c*=110.515 ± 2.028 kb. The black curve depicts the best fit obtained with the linear polymer model (Eq. 1 & 2a) (R^2 = 0.18).
