# Supplementary material for: Modulated contact frequencies at gene-rich loci support a statistical helix model for mammalian chromatin organization
Source: Genome Biol. 2011 May 10;12(5):R42. doi: 10.1186/gb-2011-12-5-r42 (PMC3219965; doi:10.1186/gb-2011-12-5-r42)
Supplement: Additional file 4 — Gene expression at loci investigated by 3C-qPCR. Total RNA from 30-day-old mouse liver was prepared and mRNA levels were determined by RT-qPCR relative to Gapdh mRNA level. The Usp22, LnP and Mtx2 genes were found to be expressed. Very low levels of expression were found for the Gtlf3b, Aldh3a2 and Emb genes. The other genes (Kcnj12, Tnfref13b, Gtl2, Dlk1 and HoxD13) are fully repressed. [file gb-2011-12-5-r42-S4.PDF]

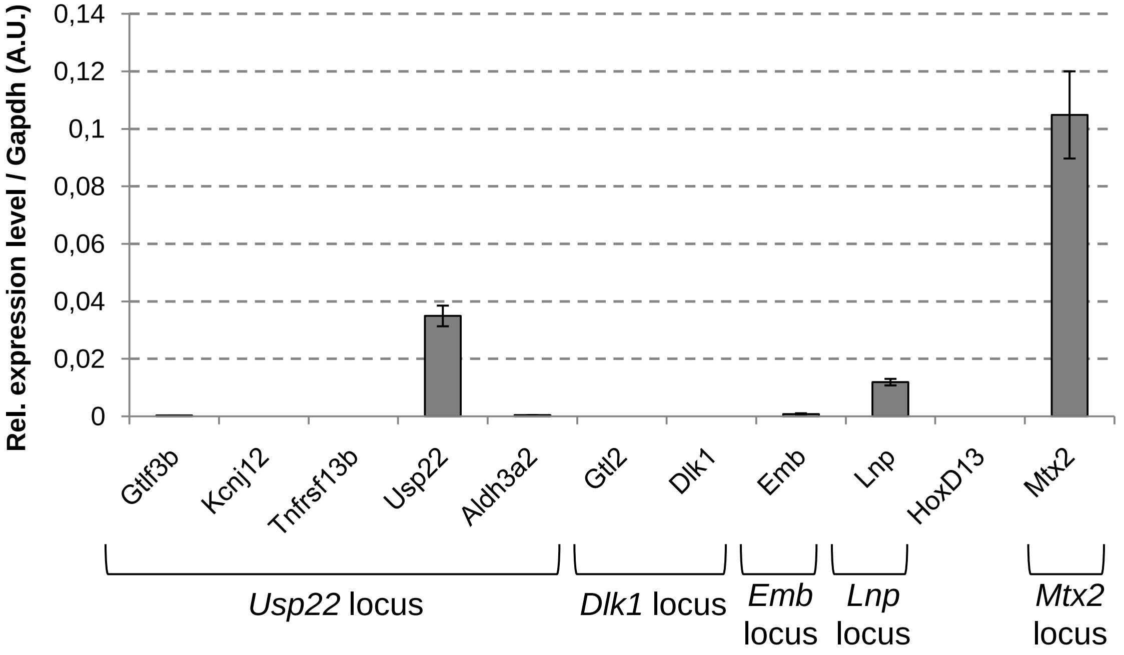

Additional data 4. Gene expression at loci investigated by 3C-qPCR. Total RNA from 30 day-old mouse liver was prepared and mRNA levels were determined by RT-qPCR relative to Gapdh mRNA level. The *Usp22, LnP* and *Mtx2* genes were found to be expressed. Very low level of expression were found for the *Gtlf3b*, *Aldh3a2* and *Emb* genes. The other genes (*Kcnj12*, *Tnfref13b*, *Gtl2*, *Dlk1* and *HoxD13*) are fully repressed.
