# Supplementary material for: Modulated contact frequencies at gene-rich loci support a statistical helix model for mammalian chromatin organization
Source: Genome Biol. 2011 May 10;12(5):R42. doi: 10.1186/gb-2011-12-5-r42 (PMC3219965; doi:10.1186/gb-2011-12-5-r42)
Supplement: Additional file 5 — Random collisions at silent versus expressed loci. Data points represent collision frequencies determined at silent (Dlk1/Emb/Lnp; black circles) or expressed (Usp22/Mtx2; red circles) loci. Best fit of the statistical helix model (Equations 1 and 5) was performed for each dataset (black curve = silent loci; red curve = expressed loci). The values of best fit parameters for each data set are indicated in the graph. Both the diameter (D) and the step (P) of the helix are larger in the expressed loci compared to the silent ones. [file gb-2011-12-5-r42-S5.PDF]

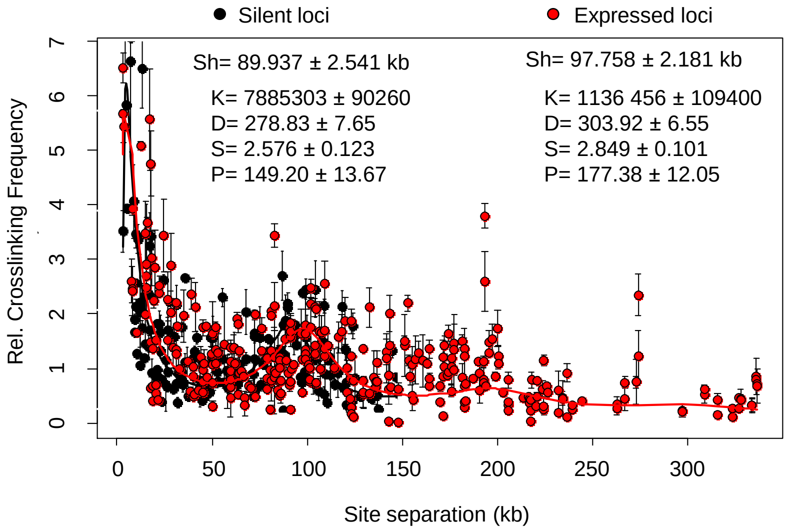

Additional data 5. Random collisions at silent *vs* expressed loci. Data points represent collision frequencies determined at silent (*Dlk1*/*Emb*/*Lnp*; black circles) or expressed (*Usp22*/*Mtx2*; red circles) loci.Best fit of the statistical helix model (Eq.1 & 5) were performed for each dataset (black curve= silent loci; red curve=expressed loci). The value of best fit parameters for each data sets are indicated in the graph. Both the diameter (D) and the step (P) of the helix are larger in the expressed loci compared to the silent ones.
