# Supplementary material for: Modulated contact frequencies at gene-rich loci support a statistical helix model for mammalian chromatin organization
Source: Genome Biol. 2011 May 10;12(5):R42. doi: 10.1186/gb-2011-12-5-r42 (PMC3219965; doi:10.1186/gb-2011-12-5-r42)
Supplement: Additional file 6 — Fitting the statistical helix model to the yeast Saccharomyces cerevisiae genome. In order to test whether a statistical helix organization may be valid for other organisms, we fitted the statistical helix polymer model to the 3C data obtained in the yeast S. cerevisiae [24]. For both AT-rich and GC-rich regions (Additional file 7a and 7b, respectively), correlation coefficients (R2 = 0.82 and 0.80, respectively) were similar to those obtained from published models (R2 = 0.81 and 0.79, respectively) [24]. For AT-rich regions, consistent with previous findings [24], the statistical helix model predicts a linear polymer organization (Additional file 7a). However, data obtained in GC-rich domains are fully compatible with a statistical helix organization. Compared to mammals, chromatin dynamics in yeast can be described as a statistical helix that would have a slightly smaller diameter (212.62 ± 31.73 nm) but a much wider step (310.94 ± 54.86) (Additional file 7b). Finally, using these best-fit parameters and Equation 4c, we calculated how, according to this statistical helix model, the spatial distances should vary as a function of genomic site separations. We found that spatial distances calculated from the statistical helix model are in good agreement with those measured in high-resolution FISH analyses performed in living yeast cells (Additional file 7c) [37]. Therefore, the statistical helix model may also be valid to describe chromatin dynamics in GC-rich domains of the S. cerevisiae genome. [file gb-2011-12-5-r42-S6.PDF]

Additional data 6 – Fitting the statistical helix model to the yeast *Saccharomyces cerevisiae* genome.

In order to test whether a statistical helix organization may be valid for other organisms, we fitted the *statistical helix* polymer model to the 3C data obtained in the yeast *Saccharomyces cerevisiae* [24]. For both AT-rich and GC-rich regions (Additional data 7A and 7B respectively), correlation coefficients (R^2 =0.82 and 0.80 respectively) were similar to those obtained from published models (R^2 =0.81 and 0.79 respectively) [24]. For AT-rich regions, consistent with previous findings [24], the statistical helix model predicts a linear polymer organization (Additional data 7A). However, data obtained in GC-rich domains are fully compatible with a statistical helix organization. Compared to mammals, in yeast, chromatin dynamics can be described as a statistical helix that would have a slightly smaller diameter (212.62 ± 31.73 nm) but a much wider step (310.94 ± 54.86) (Additional data 7B). Finally, using these best-fit parameters and equation 4c, we calculated how, according to this statistical helix model, the spatial distances should vary as a function of genomic site separations. We found that spatial distances calculated from the statistical helix model are in good agreement with those measured in high-resolution FISH (Fluorescence In Situ Hybridization) analyses performed in living yeast cells (Additional data 7C) [37]. Therefore, the statistical helix model may also be valid to describe chromatin dynamics in GC-rich domains of the *Saccharomyces cerevisiae* genome.
