# Supplementary material for: Modulated contact frequencies at gene-rich loci support a statistical helix model for mammalian chromatin organization
Source: Genome Biol. 2011 May 10;12(5):R42. doi: 10.1186/gb-2011-12-5-r42 (PMC3219965; doi:10.1186/gb-2011-12-5-r42)
Supplement: Additional file 7 — Fitting the statistical helix model to the yeast Saccharomyces cerevisiae genome. Data published by Dekker for the yeast S. cerevisiae [24] were normalized using the previously published algorithm [19] and the statistical helix polymer model (Equations 1 and 5 was fitted to normalized data. (a) For AT-rich regions, consistent with previous findings [24], the statistical helix model (red curve) predicted a linear polymer organization (black curve). In this case, the best fit values obtained for the diameter D and the step P are not relevant, as indicated by large standard deviations. (b) In GC-rich regions, the statistical helix model (red curve), fits with a distended helical shape. Best-fit parameters are indicated above the graph. They were calculated using a linear mass density of 11.1 nm/kb [11]. The black curve depicts the best fit of the linear polymer model and the green curve the best fit of the circular polymer model. Note that the lengths of the statistical fragments obtained from the statistical helix model (S = 6.060 ± 0.519 kb and 4.558 ± 0.503 kb for AT-rich and GC-rich domains, respectively) are compatible with the parameters previously obtained with the linear or circular polymer models (S = 6.4 ± 0.34 kb and 4.7 ± 0.45 kb, respectively) [24]. (b) Using the best-fit parameters obtained for the yeast S. cerevisiae (b), we calculated the expected mean spatial distances (in nm) for increasing site separation distances (0 to 140 kb) for both the statistical helix (Equation 4c; red curve) and the linear polymer (Equation 4a; black curve) models. The experimental spatial distances (in nm) obtained by Bystricky et al. (Table 1 and Supplementary Table of [37]) from high-resolution FISH experiments were plotted into this graph (open squares, adjusted average distances; black diamonds, average peak distances). The statistical helix model is in good agreement with these experimental data. [file gb-2011-12-5-r42-S7.PDF]

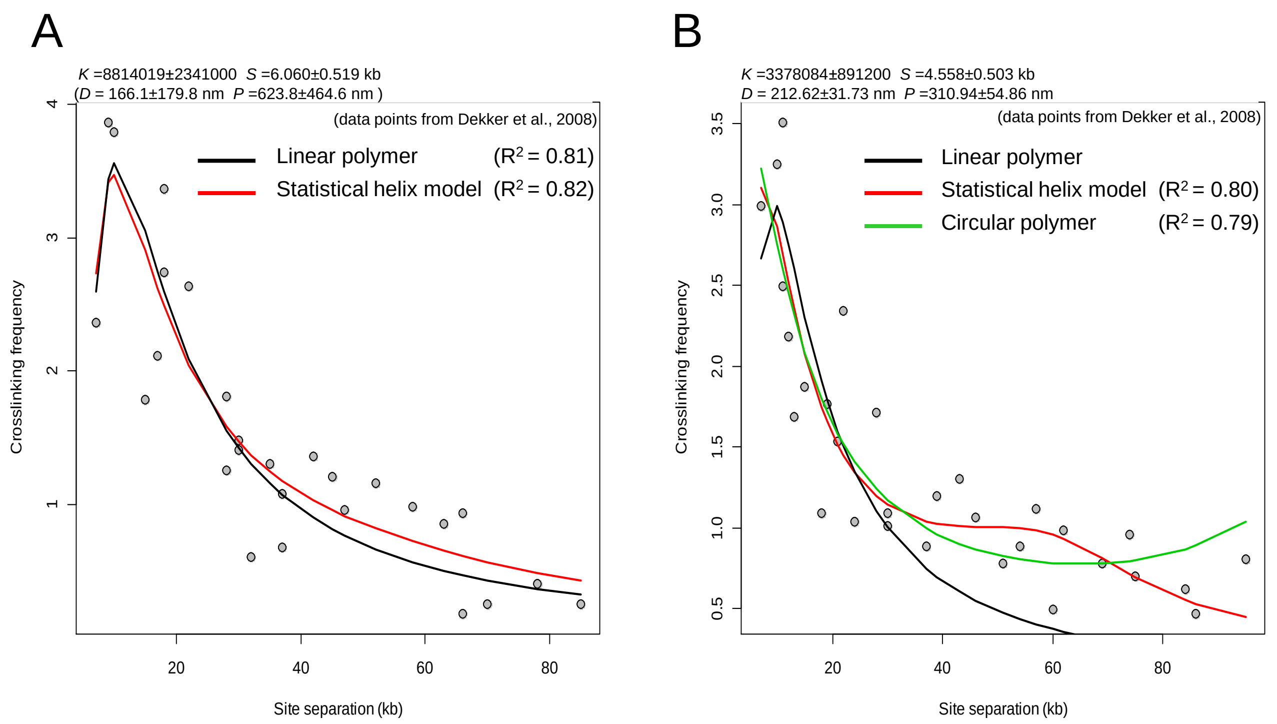

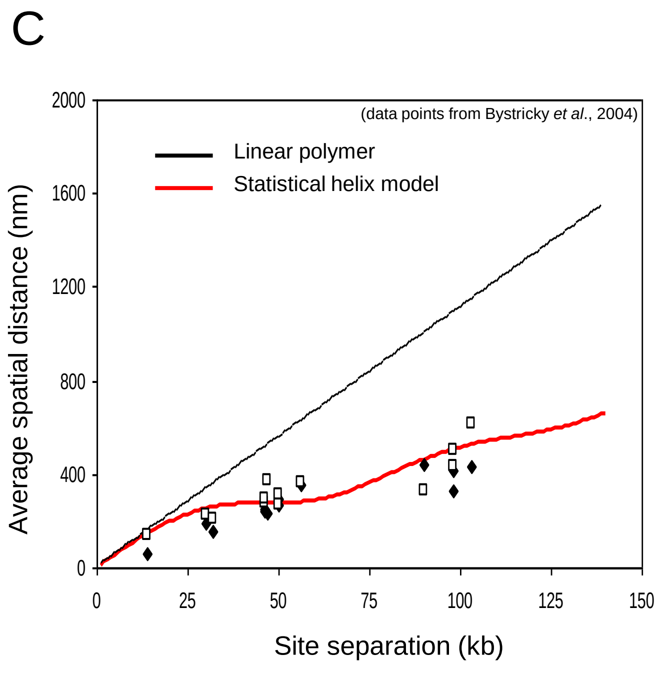

Additional data 7. Fitting the statistical helix model to the yeast *Saccharomyces cerevisiae* genome.
Data published by Dekker for the yeast *Saccharomyces cerevisiae* [24] were normalized using the previously published algorithm [19] and the statistical helix polymer model (Eq.1a & 5) was fitted to normalized data. (**A)** For AT-rich regions, consistent with previous findings [24], the statistical helix model (red curve) predicted a linear polymer organization (black curve). In this case, the best fit values obtained for the diameter *D* and the step *P* are not relevant as indicated by large standard deviations. (**B)** In GC-rich regions, the statistical helix model (red curve), fits with a distended helical shape. Best-fit parameters are indicated above the graph. They were calculated using a linear mass density of 11.1 nm/kb [11]. The black curve depicts the best fit of the linear polymer model and the green curve the best fit of the circular polymer model. Note that the lengths of the statistical fragments obtained from the statistical helix model (S = 6.060 ± 0.519 kb and 4.558 ± 0.503 kb for AT-rich and GC-rich domains respectively) are compatible with the parameters previously obtained with the linear or circular polymer models (S = 6.4 ± 0.34 kb and 4.7 ± 0.45 kb respectively) [24]. (**C**) Using the best-fit parameters obtained for the yeast *Saccharomyces cerevisiae* (panel B), we calculated the expected mean spatial distances (in nm) for increasing site separation distances (0 to 140 kb) for both the statistical helix (Eq.4c; red curve) and the linear polymer (Eq.4a; black curve) models. The experimental spatial distances (in nm) obtained by Bystricky *et al.* (Table 1 & supplementary table 4 of reference [37]) from high-resolution FISH experiments were plotted into this graph (open squares : adjusted average distances and black diamonds: average peak distances). The statistical helix model is in good agreement with these experimental data.
