# Supplementary material for: Modulated contact frequencies at gene-rich loci support a statistical helix model for mammalian chromatin organization
Source: Genome Biol. 2011 May 10;12(5):R42. doi: 10.1186/gb-2011-12-5-r42 (PMC3219965; doi:10.1186/gb-2011-12-5-r42)
Supplement: Additional file 8 — An upper limit of validity for the statistical helix model. Expected spatial distances (in nm) were calculated as a function of increasing genomic distances (in kb) using either Equation 4a (linear polymer model, black curve, with L = 9.6 nm/kb) or Equation 4c and the biophysical parameter given in Figure 4 (statistical helix model, red curve). Dashed lines represent the expected deviations due to standard errors on the measured biophysical parameters (Figure 4). Details about mathematical equations are given in the Materials and methods section. Data points (blue diamonds) depict spatial distances measured by FISH experiments as reported by van den Engh et al. [32]. These data points were obtained from a gene-rich chromosomal region containing the Huntington disease locus. [file gb-2011-12-5-r42-S8.PDF]

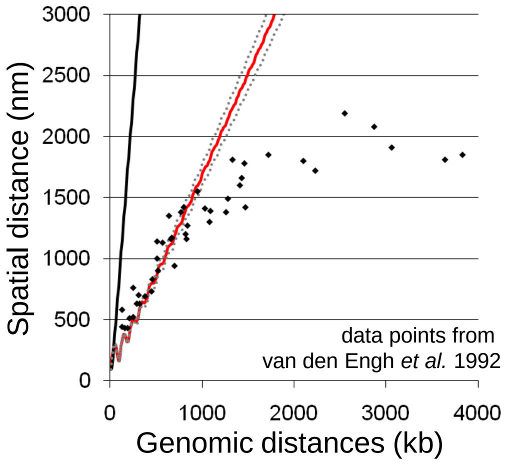

Additional data 8. An upper limit of validity for the statistical helix model.

Expected spatial distances (in nm) were calculated as a function of increasing genomic distances (in kb) using either the Eq.4a (linear polymer model, black curve, with *L*=9.6 nm/kb) or the Eq.4c and the biophysical parameter given in Fig.4 (statistical helix model, red curve). Dashed lines represent the expected deviations due to standard errors on the measured biophysical parameters (Fig.4). Details about mathematical equations are given in the methods section. Data points (blue diamonds) depict spatial distances measured by FISH experiments as reported by Ger van den Engh *et al.* [32]. These data points have been obtained from a gene-rich chromosomal region containing the Huntington disease locus.
